# Heterogeneity in Brain Microstructural Development Following Preterm Birth

**DOI:** 10.1093/cercor/bhaa069

**Published:** 2020-04-18

**Authors:** Ralica Dimitrova, Maximilian Pietsch, Daan Christiaens, Judit Ciarrusta, Thomas Wolfers, Dafnis Batalle, Emer Hughes, Jana Hutter, Lucilio Cordero-Grande, Anthony N Price, Andrew Chew, Shona Falconer, Katy Vecchiato, Johannes K Steinweg, Olivia Carney, Mary A Rutherford, J-Donald Tournier, Serena J Counsell, Andre F Marquand, Daniel Rueckert, Joseph V Hajnal, Grainne McAlonan, A David Edwards, Jonathan O’Muircheartaigh

**Affiliations:** 1 Centre for the Developing Brain, Department of Perinatal Imaging and Health, School of Biomedical Engineering and Imaging Sciences, King’s College London, London, SE1 7EH, UK; 2 Department for Forensic and Neurodevelopmental Sciences, Institute of Psychiatry, Psychology and Neuroscience, King’s College London, London, SE5 8AF, UK; 3 Department of Electrical Engineering, ESAT/PSI, KU Leuven, Leuven, 3001, Belgium; 4 Donders Centre for Cognitive Neuroimaging, Donders Institute for Brain, Cognition and Behaviour, Radbound University, Nijmegen, 6525EN, the Netherlands; 5 Department of Cognitive Neuroscience, Radbound University Medical Centre, Nijmegen, 6525EN, the Netherlands; 6 Biomedical Image Technologies, ETSI Telecomunicacion, Universidad Politecnica de Madrid and CIBER-BBN, Madrid, 28040, Spain; 7 Biomedical Image Analysis Group, Department of Computing, Imperial College London, London, SW7 2AZ, UK; 8 MRC Centre for Neurodevelopmental Disorders, King’s College London, London, SE1 1UL, UK; 9 South London and Maudsley NHS Foundation Trust, London, SE5 8AZ, UK

**Keywords:** biological heterogeneity, early brain development, neonatal MRI, neurodevelopment, prematurity

## Abstract

Preterm-born children are at increased risk of lifelong neurodevelopmental difficulties. Group-wise analyses of magnetic resonance imaging show many differences between preterm- and term-born infants but do not reliably predict neurocognitive prognosis for individual infants. This might be due to the unrecognized heterogeneity of cerebral injury within the preterm group. This study aimed to determine whether atypical brain microstructural development following preterm birth is significantly variable between infants. Using Gaussian process regression, a technique that allows a single-individual inference, we characterized typical variation of brain microstructure using maps of fractional anisotropy and mean diffusivity in a sample of 270 term-born neonates. Then, we compared 82 preterm infants to these normative values to identify brain regions with atypical microstructure and relate observed deviations to degree of prematurity and neurocognition at 18 months. Preterm infants showed strikingly heterogeneous deviations from typical development, with little spatial overlap between infants. Greater and more extensive deviations, captured by a whole brain atypicality index, were associated with more extreme prematurity and predicted poorer cognitive and language abilities at 18 months. Brain microstructural development after preterm birth is highly variable between individual infants. This poorly understood heterogeneity likely relates to both the etiology and prognosis of brain injury.

## Introduction

Preterm birth affects approximately 10% of all live births worldwide (Chawanpaiboon et al. 2019) and represents a leading cause of infant mortality and morbidity (Liu et al. 2015). Prematurity increases the risk of atypical brain development and has been associated with a wide range of neurocognitive and behavioral deficits that often persist into adolescence. These include higher incidence of attention-deficit/hyperactivity disorder (ADHD) and autism spectrum disorder among survivors (Johnson and Marlow 2011; Bhutta et al. 2014; Agrawal et al. 2018). Gestational age (GA) at birth is important but is not the sole determinant of later functional outcome. Instead, enormous clinical diversity of individuals born preterm likely reflects multifactorial causes which have heterogeneous effects on brain development.

Diffusion MRI has been used to characterize the cerebral consequences of prematurity and assign neurocognitive prognosis. Studies of preterm infant at a group level have identified important neuroanatomical correlates of the various behavioral impairments in this population. As a group, preterm infants show atypical maturation at term-equivalent age compared with term-born infants (van Kooij et al. 2012; Ball et al. 2013, 2015) that has been related to poorer neurodevelopmental outcome (Counsell et al. 2008; van Kooij et al. 2012; Duerden et al. 2015). Prematurity has been associated with subtle diffuse microstructural alterations, where preterm infants show microstructural diffusion consistent with more “immature” white and gray matter compared with term-born infants (Counsell et al. 2003; Anjari et al. 2007; Ball et al. 2013).

However, studies comparing group means assume that groups are homogenous, for example, in the location and the extent of abnormalities, and do not allow for different alterations in different individuals within the same group. Given the highly variable clinical courses suffered by preterm infants, such homogeneity cannot be assumed. We hypothesized that individual heterogeneity may in part be the cause for relatively poor predictive power of neonatal MRI, reasoning that this may account for why neither conventional nor diffusion MRI are precise predictors of individual neurocognitive prognosis, particularly in the absence of focal major lesions (de Bruïne et al. 2011; Mürner-Lavanchy et al. 2019).

However, capturing abnormal brain maturation at an individual level in this vulnerable cohort is a considerable challenge (Batalle et al. 2018). To address this, we adopted a nonparametric model estimation technique, Gaussian process regression (GPR), to define normative microstructural development on a voxel level using diffusion MRI. This method produces a “mapping” of normal development and allows a comparison of each brain voxel in individual infants to normative values. The approach is analogous to pediatric growth charts where individual observations are compared in reference to a normative distribution (typical variation) and presented as a standardized deviation (*Z*-score) from the expected mean given the age and sex of the individual.

Using a large sample of term-born infants, we estimated a normative model which provided normal ranges for a number of diffusion measures at every voxel in the brain. We then quantified the voxel-level variation in brain microstructural development for individual preterm infants at term-equivalent age. We predicted that preterm infants would show a high degree of heterogeneity in the extent and spatial distribution of deviations from typical development and exhibit higher overall proportion of brain voxels deviating compared with term-born infants. To ensure that any extreme deviations are relevant to brain development and function, we related a summary measure of abnormality, an atypicality index, to the degree of prematurity and neurodevelopment at 18 months.

## Material and Methods

### Participants

Infants were recruited and imaged for the developing Human Connectome Project (http://www.developingconnectome.org/), approved by the National Research Ethics Committee (REC: 14/LO/1169). Informed written parental consent was obtained prior to imaging. Infants were scanned during natural unsedated sleep. Pulse oximetry, respiratory rate, electrocardiography, and temperature were monitored throughout the scan. Ear protection included earplugs molded from a silicone-based putty (President Putty, Coltene Whaledent) placed in the external auditory meatus and neonatal earmuffs (MiniMuffs, Natus Medical Inc.).

The final study sample consisted of 352 (270 term-born) infants (Table 1) scanned after 37 weeks postmenstrual age (PMA). All MRI images were examined by neonatal neuroradiologists, and no infants with major brain lesions (cerebral infarctions) or congenital abnormalities were included in the term-born group. While the incidence of punctate white matter lesions (PWMLs) and their relation to later neurodevelopment have been studied in preterm infants (Tusor et al. 2017), the occurrence and the long-term consequences of PWMLs in healthy term-born infants remains unknown, due to the lack of large cohort studies in this population. Therefore, the presence of PWMLs was not an exclusion criterion. There were no exclusion criteria for the preterm sample.

**Table 1:** Perinatal and neurocognitive characteristics of term and preterm infants

	Term-born *n* = 270	Preterm-born *n* = 82	*P* value
Gestational age (weeks), median (IQR)	40 (39–40.9)	32.6 (29.6–35.1)	—
Postmenstrual age (weeks), median (IQR)	40.8 (39.4–42)	41 (39.6–42.3)	*P* = 0.53
Female sex, no. (%)	133 (49%)	40 (49%)	*P* > 0.99
Scan HC (cm), mean ± SD	34.7 ± 1.5	34.9 ± 1.8	*P* = 0.36
Birth HC (cm), mean ± SD	34.3 ± 1.5	29.5 ± 3.6	*P* < 0.005
Birth weight (kg), mean ± SD	3.31 ± 0.5	1.8 ± 0.76	*P* < 0.005
APGAR score 1 min, median (IQR)	9 (8–9)	7 (5–9)	*P* < 0.005
APGAR score 5 min, median (IQR)	10 (9–10)	9 (8–10)	*P* < 0.005
PWMLs, no. infants (%)	36 (13%)	32 (39%)	*P* < 0.05
BSID-III, no. infants (%)	210 (78%)	47 (57%)	—
Age (months), median (IQR)	18.4 (18–18.7)	18.4 (18–19)	*P* = 0.87
Gestation age (weeks), median (IQR)	40 (39–40.9)	31.9 (29.1–35.9)	—
Postmenstrual age (weeks), median (IQR)	41.1 (39.4–41.8)	41.1 (39–42.6)	*P* = 0.61
Female sex, no. (%)	106 (50%)	24 (51%)	*P* > 0.99
BSID-III motor, mean ± SD	101.6 ± 9.8	99.3 ± 11.5	*P* = 0.2
BSID-III cognitive, mean ± SD	100 ± 11	101 ± 12.7	*P* = 0.3
BSID-III language, mean ± SD	96.3 ± 15	98.5 ± 16.4	*P* = 0.23
IMD score, median (IQR)	26.9 (17–36.4)	17.4 (10–29.6)	*P* < 0.005
PWMLs, no. infants (%)	33 (16%)	19 (40%)	*P* < 0.005

Notes: IQR, interquartile range; APGAR, appearance, pulse, grimace, activity, and respiration. Missing data: scan HC (2 term/2 preterm), birth HC (17 term/9 preterm); birth weight (1 term); APGAR score (32 term/7 preterm); IMD (13 term/3 preterm).

The Bayley III Scales of Infant and Toddler Development (BSID-III; Bayley 2006) collected at 18 months were available for 257 (210 term) infants. The BSID-III assesses motor, cognitive, and language development. Here we used the composite scores of all three scales, ranging between 40 and 160 with a mean/standard deviation (SD) of 100/15. Assessments were carried out by developmental pediatricians/psychologists experienced in administering neurocognitive assessments in toddlers.

### Diffusion MRI Acquisition and Preprocessing

dMRI data were acquired on a 3T Philips Achieva scanner equipped with a dedicated 32-channel neonatal head coil and baby transportation system (Hughes et al. 2017). A total of 300 volumes were acquired sampling *b*-values of 400, 1000, and 2600 s/mm^2^ spherically distributed in 64, 88, and 128 directions, together with 20 *b* = 0 s/mm^2^ images. Acceleration of multiband 4, SENSE factor 1.2, and partial Fourier 0.86 were used, acquired resolution 1.5 × 1.5 mm, 3 mm slices with 1.5 mm overlap, TR/TE of 3800/90 ms, and 4 phase-encoding directions. Total acquisition time for the dMRI protocol was 20 min. Protocol optimization for the dMRI data collected as part of the dHCP is described in details in Hutter et al. (2018).

dMRI data were preprocessed with denoising (Veraart et al. 2016), Gibbs ringing removal (Kellner et al. 2016), and *B*_0_ field map estimation (Andersson et al. 2003) and reconstructed using a slice-to-volume motion and distortion correction with slice-level outlier rejection using a multishell spherical harmonics and radial decomposition signal representation (Christiaens et al. 2019). This resulted in images with isotropic resolution of 1.5 mm. Visual inspection confirmed that motion correction and outlier rejection were successful, and data of insufficient quality were excluded. A summary motion metric was quantified using the total infant translation, rotation, and the ratio of detected outliers following the procedure described in Christiaens et al. (2019) (see Supplementary Materials). The tensor model was fitted to the *b* = 400 and *b* = 1000 s/mm^2^ shells. Scalar maps of fractional anisotropy (FA), quantifying the degree of anisotropy within a voxel, and mean diffusivity (MD), in μm^2^/ms (10^−3^ mm^2^/s), reflecting the overall magnitude of diffusion, were calculated using MRtrix3 (Tournier et al. 2019).

Prior to voxel-wise analysis, we applied a customized processing pipeline specifically developed for the neonatal dMRI data collected as part of the dHCP described in detail in Pietsch et al. (2019). In summary, for each infant, the diffusion-weighted signal was decomposed into isotropic fluid and anisotropic tissue (GM-WM) components (see Supplementary Fig. 1) using subject-specific response functions estimated from CSF and WM via multishell multitissue constrained spherical deconvolution (MSMT-CSD; Jeurissen et al. 2014). For each infant, the estimated orientation distribution functions (ODFs) were subsequently intensity normalized and bias field corrected (mtnormalise, MRtrix3). The tissue and fluid ODFs were registered to the age-matched (PMA at scan—37.1, 38.1, 39.1, 40.1, 40.9, 42.0, 42.8, and 44.1 weeks) and coaligned templates (Pietsch et al. 2019; visualization of one of the weekly templates is provided in Supplementary Fig. 1) using a multicontrast affine and nonlinear transformation model that takes the appropriate reorientation of the ODFs into account (mrregister, MRtrix3). Similar to other state-of-the-art registration algorithms such as DTI-TK (Zhang et al. 2006), the multicontrast ODF registration framework applied here benefits from taking into account the underlying orientation structure in every voxel. This approach has been shown to provide higher registration accuracy and “sharper features” in spatial/angular domain (Pietsch et al. 2017). The templates will be available from http://brain-development.org/brain-atlases/.

The resulting deformation fields were used to transform the scalar tensor maps into the common space. Satisfactory spatial alignment to template space with no gross misalignments was confirmed by a visual examination of the transformed maps. After subject-wise concatenation, this resulted in two sets of 4D images (one per tensor metric per group) which served as input to the GPR.

### Gaussian Process Regression

To characterize normative microstructural development, we used multioutput GPR (Alvarez et al. 2012), implemented in GPy, a Gaussian processes framework in python (https://sheffieldml.github.io/GPy/). GPR is a Bayesian nonparametric model estimation technique that simultaneously provides coherent measures of predictive confidence in addition to point estimates. The predictive confidence is used as an uncertainty measure that represents the deviation of each infant from the normative group mean on a voxel level. This can be statistically quantified as a *Z*-score by computing the difference between the predicted and the observed value normalized by the uncertainty of the prediction (Marquand et al. 2016).

First, we estimated the normative variation within the term-born (training) sample using FA and MD maps as inputs and PMA at scan and sex as predictors. The GPR was estimated separately for every voxel, simultaneously modelling FA and MD to take advantage of the shared information between the measures. The accuracy of the model was estimated under 5-fold cross-validation, where folds were stratified so that each subset covered the whole range of PMA in the study, ranging from 37 to 45 weeks. The association between brain microstructure and the model predictors was estimated with a combination of radial basis function and white noise covariance kernels. Model hyperparameters were optimized using log marginal likelihood. The voxel-level prediction performance of the model was evaluated using the mean absolute error (MAE) between the predicted and the observed values from the leave-one-out 5-fold cross-validation.

The final model, trained on the whole term sample, was subsequently applied to the preterm (holdout) sample. Individual *Z*-score maps were computed for both metrics. Extreme deviations from the normative model were calculated by applying a threshold of |*Z*| > 3.1. Extreme positive deviations, hereafter also referred to as FA+/MD+, indicated that the observed value was lying more than 3.1 SD above the predicted mean for the age and sex of the infant, while extreme negative deviations, henceforth FA−/MD−, indicated that the observed value was more than 3.1 SD lower than the predicted mean. The spatial overlap of extreme deviations within the groups was assessed by calculating a voxel-wise percentage map of extreme deviation (number of subjects with |*Z*| > 3.1 divided by the total number of subjects). We examined the voxels where more than 4% of the preterm group showed extreme deviations from the normative model and assessed normal extreme variability by defining voxels with extreme deviations in more than 4% of the infants in the term group.

The python code used to run voxel-wise GPR as applied in this work is available at (http://www.github.com/jonnyomuir).

### Whole Brain Atypicality Index

Four whole brain atypicality indices were calculated to capture the extreme deviations in both directions separately, FA+, FA−, MD+, and MD− for each infant, computed as the percentage of voxels with extreme deviations (|*Z*| > 3.1) relative to the total number of intracerebral (Wolfers et al. 2018, 2019). We investigated the association between prematurity and this atypicality index by 1) calculating the correlation with GA at birth (Spearman rho, ρ) and 2) investigating whether the group-average atypicality index is higher in preterm compared with term infants (Mann–Whitney *U*; Vargha and Delaney’s *A* effect size estimate (Vargha and Delaney 2000); Supplementary Materials). Multiple comparison correction was applied using the Bonferroni–Holms method (Holm 1978). Analyses were performed in R 3.4.4 (www.r-project.org) and visualized in python 3.7 (www.python.org).

### Association Between Neonatal Microstructure and Later Neurodevelopment

The association between the atypicality index and Bayley’s scores was assessed using Spearman ρ. To further examine significant associations, we tested whether atypicality indices were predictive of behavior when entered into multiple regression models together with GA at birth, sex, the aggregate measure of head motion, and socioeconomic score, captured by the English Index of Multiple Deprivation (IMD). The IMD provides a summary measure considering 38 different factors including income, employment, education, health, and crime for every area in England, using national census data. Higher IMD score relates to higher level of deprivation. Assessment of the assumptions of the linear model were carried out by testing the normality of the models’ residuals and using the glvma package in R (Peña and Slate 2012), assessing global statistics is the relationship between the predictors and the outcome linear, skewness, kurtosis, link function, and heteroscedasticity. Furthermore, for every model we generated bootstrapped 95% confidence intervals (CI, 1000 replications using boot package in R (Davison and Hinkley 1997) for the adjusted *R*^2^ and used bootstrap resampling (*n* = 100) to evaluate the accuracy of the prediction by using the adjusted *R*^2^ averaged across all resamples.

## Results

### Sample Characteristics

There were no differences in PMA at scan, the proportion of male/female between term and preterm infants, nor did the two samples differ in head circumference (HC) at scan (Table 1). As expected, preterm infants had lower birth weight and smaller birth HC compared with full-term infants. PWMLs were more frequently observed in the preterm sample but were present in 13% of the term-born infants. Out of the 82 preterm infants included, one had a major incidental finding of cerebellar hemispheric parenchymal loss accompanied by brainstem atrophy, one had two small focal hemorrhages in the left cerebellar hemisphere, and one had small focal hemorrhages in the cerebellar hemispheres bilaterally.

Between the term and preterm infants with available neurodevelopmental follow-up at 18 months, group differences were evident in incidence of PWMLs and IMD scores, with preterm infants showing significantly lower deprivation (Table 1). Association between neurodevelopment and GA at birth was found only in the preterm sample, indicating higher GA at birth is associated with better cognitive score (ρ = 0.38, *p*_corr_ < 0.05; see Supplementary Table 1). Higher IMD score, or higher deprivation, was related to lower cognitive performance in the term group alone (ρ = −0.26, *p*_corr_ < 0.005) and no association between deprivation and neurodevelopment was found in the preterm sample (see Supplementary Table 2).

**Figure 1:**
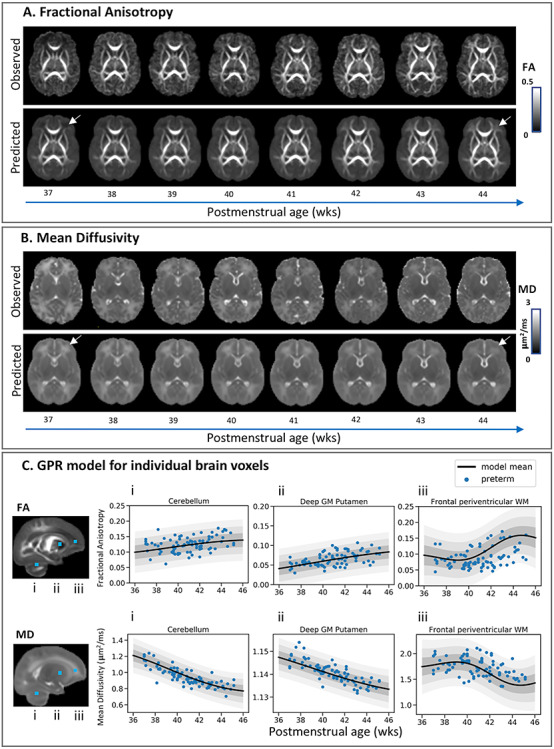
Modelling the developing brain microstructure using Gaussian process regression. Observed and predicted individual infant fractional anisotropy (*A*) and mean diffusivity (*B*) maps for eight full-term infants randomly selected to represent every week between 37 and 44 weeks PMA. The effect of PMA is best seen in the frontal periventricular WM, highlighted by the white arrows. The predicted developmental trajectories are plotted for three randomly selected brain voxels (*C*) located in the cerebellum (*i*), deep GM putamen (*ii*), and frontal periventricular WM (*iii*). The relative location of the voxels is highlighted with blue squares on the observed mean FA and MD maps. Plots show the model mean (thick black) ±1 (dark gray), ±2 (light gray), and ±3 (lighter gray) standard deviations from the predicted mean and the diffusion values extracted for these voxels for all 82 preterm infants.

### Modelling the Developing Neonatal Microstructure Using GPR

Figure 1 depicts example images of the observed and predicted individual infant FA and MD maps. Qualitatively, the effects of PMA were most noticeable in the periventricular white matter (WM), where FA increased (dark to bright) and MD decreased (bright to dark). Prediction was sharper in the subcortical and central WM tracts and blurrier in the cortex, a problematic area due to the thin nature of the structure and the high intrasubject anatomical variability (Dubois et al. 2016; Bozek et al. 2018), posing a significant challenge for image registration. Examples of the normative model for three randomly selected voxels (cerebellum, frontal periventricular WM, and putamen) are shown in Figure 1*C*, along with mapping of preterm infants to the normative curves (the term data are shown in Supplementary Fig. 2). The whole brain average MAE was 0.031 for FA and 0.17 μm^2^/ms for MD (see Supplementary Fig. 3). The MAE for the cerebellum was 0.0053 μm^2^/ms for MD and 0.0181 for FA, for periventricular WM 0.12 μm^2^/ms for MD and 0.0219 for FA, and for putamen 0.031 μm^2^/ms for MD and 0.0135 for FA.

### Characterizing the Heterogeneous Effect of Prematurity on the Developing Microstructure

**Figure 2:**
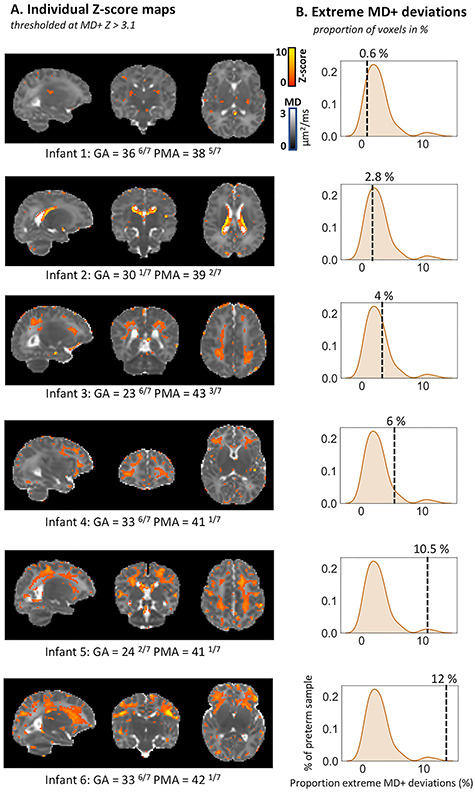
Detecting deviations from normative development at the level of the individual preterm infant. Individual MD+ deviations (*Z*-score maps thresholded at *Z* > 3.1) are shown for six preterm infants depicting the unique spatial patterns observed within the preterm sample (*A*). The density plots (*B*) indicate where is the overall brain proportion of extreme deviations for this infant in relation to the rest of the preterm sample. Infants were selected to show different overall proportions of extreme deviations. Infant 1 had a relatively low atypicality index (0.6% of voxels), while infant 6 had a relatively high index (12%) of voxels deviating from the model. The GA at birth and PMA at scan (in weeks ^+days^) are also shown for each infant.

In preterm infants extreme FA deviations were widely distributed throughout the brain, with consistent abnormality found only in small clusters of voxels. MD changes were also widely distributed but with somewhat greater consistency. Figure 2 highlights the intrasubject heterogeneity of MD+ deviations observed within the preterm sample. Individual MD+ *Z*-score maps for the term sample are shown in Supplementary Figure 4. The changes were seen predominantly in regions adjacent to the ventricles, in periventricular WM, and in the periphery of the corticospinal projections to the somatosensory and motor cortex and were of varying extent and spatial distribution across infants. Clusters of MD− deviations were seen in the cerebellum, more often in the medial and superior portions, yet again with very little spatial overlap across individual subjects (see Supplementary Fig. 5). MD− clusters were found in the posterior periventricular WM indicative of PWMLs (see Supplementary Fig. 6). On other occasions, individual MD− deviations were scattered single or small collection of voxels in the periphery of the brain possibly resulting from individual differences in cortical anatomy, not resolved by registration, voxels containing nonbrain tissue, or partial volume effects.

### Spatial Overlap of Extreme Deviations Across the Developing Brain

**Figure 3:**
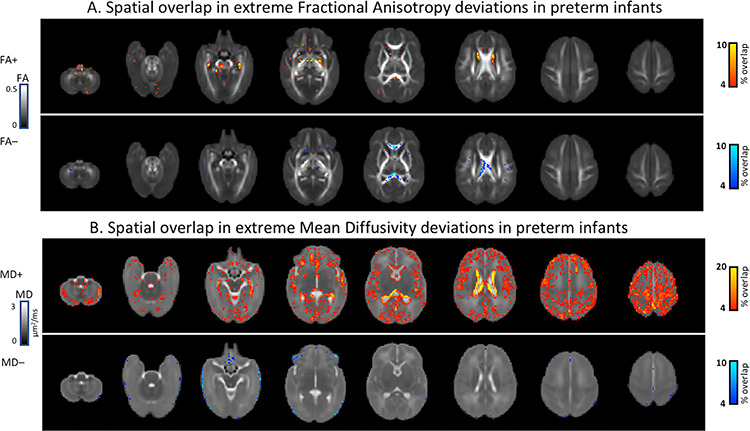
Percentage spatial overlap of extreme deviations across the developing preterm and term brain imaged at term-equivalent age. Spatial overlap in fractional anisotropy (*A*) and mean diffusivity (*B*) maps depicting areas of the brain where more than 4% of the preterm sample or more than three infants have extreme deviations from the model mean given their age and sex.

The only brain regions where more than three preterm infants (4%) showed extreme FA deviations from normative ranges were the caudate (FA+) and the corpus callosum (FA−). In all other voxels fewer than three infants had extreme deviations (Fig. 3*A*). The spatial overall in MD+ was somewhat more spread with extreme deviations in more than three infants found in voxels located in the cortex and the WM, yet only regions adjacent to the ventricles indicated an overlap in more than eight preterm infants (>10%). Upon examination of the individual *Z*-score MD+ maps, we found that 13 out of the 82 preterm infants had extreme values in voxels located around the ventricles characterized by high variability in the location and the extent of the deviations (see Supplementary Fig. 7). Very few voxels showed spatial overlap in MD− deviations, and these were primarily seen in the periphery of the brain. On average, term infants did not deviate substantially from the normative model and showed fewer extreme deviations (see Supplementary Fig. 8).

### Association Between Neonatal Brain Atypicality Index and Prematurity

**Figure 4:**
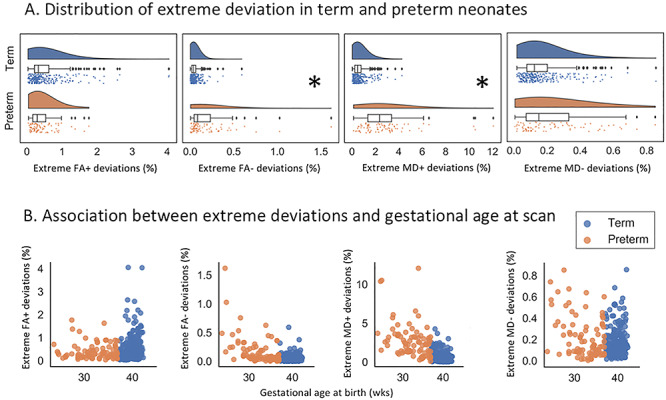
Association between prematurity and proportion of extreme deviations, captured by the atypicality index. (*A*) Preterm infants had higher FA− and MD+ atypicality indices compared with term-born infants (highlighted by a star). There was no difference between the two samples in the proportion FA+ and MD−. (*B*) Associations between the atypicality index and GA at birth in term and preterm infants. In the preterm sample, there was a negative association between GA at birth and FA− and between GA at birth and MD+ deviations. Note that the *y*-axis in *B* is not fixed.

There was no difference between term and preterm infants in the FA+ (median [IQR] − 0.33% [0.22–0.62] and 0.29% [0.17–0.54], respectively, *p*_corr_ = 0.24) and MD− atypicality indices (0.12% [0.08–0.2] and 0.15% [0.07–0.33], respectively, *p*_corr_ = 0.15). However, preterm infants had higher MD+ atypicality index (2.34% [1.35–3.4]) compared with term-borns (0.47% [0.23–0.77], *p*_corr_ < 0.005; large effect size, *A* = 0.9). Furthermore, FA− atypicality index was higher in the preterm (0.08% [0.05–0.23]) than in the term group (0.04% [0.02–0.06], *p*_corr_ < 0.005; medium effect size, *A* = 0.75; Fig. 4*A*).

Within the preterm group, GA at birth was negatively correlated with FA− (ρ = −0.49, *p*_corr_ < 0.005) and MD+ (ρ = −0.44, *p*_corr_ < 0.005) deviations. No association was observed between GA and FA+ (ρ = 0.006, *p*_corr_ = 0.99) or MD− (ρ = −0.25, *p*_corr_ = 0.09) deviations (Fig. 4*B*). Within the term sample, we found a correlation between GA and extreme FA+ (ρ = 0.28, *p*_corr_ < 0.005) and extreme MD+ deviations (ρ = −0.20, *p*_corr_ < 0.005) and no association with extreme FA− (ρ = 0.09, *p*_corr_ = 0.43) and MD− (ρ = 0.04, *p*_corr_ = 0.99).

### Association Between Neonatal Brain Atypicality Index and Neurodevelopment at 18 Months

**Figure 5:**
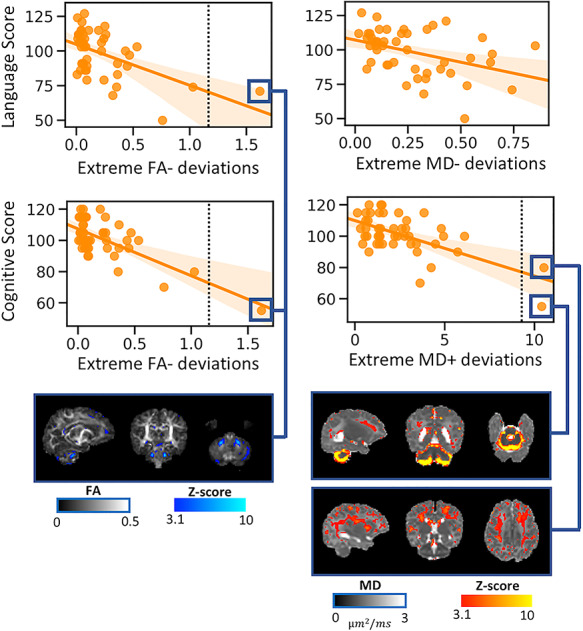
Associations between whole brain atypicality index in preterm neonates scanned at term-equivalent age and neurodevelopment at 18 months. Language scores correlated with FA− and MD− atypicality indices, while cognitive performance was associated with FA− and MD+ atypicality indices. The higher the overall loading of extreme deviations from the normative model, the lower the score at 18 months. The figure also shows the individual *Z*-score maps for three influential observations (|*Z*| > 3.1, as indicated by the vertical line on the scatterplots).

There was no significant correlation between the atypicality indices and the Bayley scales in the term-born infants (see Supplementary Table 3). However, in the preterm group, we found an association between language abilities and the overall proportion of FA− and MD− deviations (ρ = −0.46, *p*_corr_ = 0.012, and ρ = −0.40, *p*_corr_ = 0.047; Fig. 5). Furthermore, cognitive performance was associated with the overall proportion of FA− and MD+ deviations (ρ = −0.47, *p*_corr_ = 0.011, and ρ = −0.41, *p*_corr_ = 0.047; Fig. 5). To assess the effect of influential datasets on these significant correlations, we converted the atypicality indices to *Z*-scores and reran the analyses removing observations of |*Z*| > 3.1. One infant was excluded for FA−, two for MD+, and none for MD− atypicality indices (highlighted in Fig. 5). While the correlations between FA− atypicality index and neurocognition remained significant (language: ρ = −0.426, *p*_corr_ = 0.038; cognition: ρ = −0.432, *p*_corr_ = 0.033), the correlation between MD+ atypicality index and cognitive score did not survive after multiple comparison correction, although it remained of medium effect size (ρ = −0.32, *p*_corr_ = 0.37).

A linear model with FA− atypicality index (*p* < 0.005), GA (*p* = 0.26), sex (*p* = 0.30), IMD (*p* = 0.33), and motion (*p* = 0.8) explained 23% of the variance in language score (adj. *R*^2^ [95% CI] = 0.248 [0–0.34]; *F*_5,38_ = 3.6, *P* = 0.009; bootstrapped *R*^2^ = 0.18). A linear model with MD− atypicality index (*p* = 0.04), GA (*p* = 0.84), sex (*p* = 0.72), motion (*p* = 0.35), and IMD (*p* = 0.50) explained 7% of the variance in language score; however, this model was not significant (adj. *R*^2^ [95% CI] = 0.07 [−0.09 to 0.17]; *F*_5,38_ = 1.67, *P* = 0.16; bootstrapped *R*^2^ = 0.068). Fifty percent of the variance in cognitive score at 18 months was explained by FA− atypicality index (*p* < 0.005), GA (*p* = 0.62), sex (*p* = 0.07), motion (*p* = 0.58), and IMD (*p* = 0.32) (adj. *R*^2^ [95% CI] = 0.496 [0.09–0.75]; *F*_5,38_ = 9.46, *p* < 0.005; bootstrapped *R*^2^ = 0.386). MD+ atypicality index (*p* < 0.005), GA (*p* = 0.42), sex (*p* = 0.14), motion (*p* = 0.40), and IMD (*p* = 0.19) explained 39% of the variance in cognitive score (adj. *R*^2^ [95% CI] = 0.387 [0.09–0.66]; *F*_5,38_ = 76.4, *P* < 0.005; bootstrapped *R*^2^ = 0.28).

## Discussion

This study combined a large dataset of neonatal MRI from term-born infants with an innovative normative modelling technique to characterize typical variation of quantitative microstructural measurements at every brain voxel. This allowed brain images from individual infants to be compared with the normative range at a voxel level. Preterm infants showed significant deviations from typical development, but these deviations were heterogeneous in both their extent and spatial distribution, with little overlap in brain regions between infants. These deviations signal functionally significant abnormality, as more preterm infants showed greater atypicality, and the degree of deviation was related to cognitive and language performance at 18 months.

The variability in extreme deviations from normative development was striking, with greatest deviations seen in MD+. The maturation of brain tissue during early postnatal life comprises an increase in cell density, complexity, and myelination together with a reduction in tissue water content. The combination of several or all of these factors is believed to mediate the association between advancing age and the fall in MD. Therefore, the higher loading of MD+ deviations seen in preterm infants could be associated with overall more “immature” state of the brain microstructure in preterm compared with typical development. However, this microstructural abnormality does not occur in similar brain regions in all infants.

The greatest consistency in preterm MD+ deviations was in the regions adjacent to the lateral ventricles, but even in this region only 13 of 82 infants showed significant abnormalities. Enlargement of the ventricles and alterations to their shape and relative position are often observed in preterm infants imaged at term-equivalent age (Brouwer et al. 2012; Paquette et al. 2017). These, combined with other brain morphological alterations such as dolichocephalic head configuration (McCarty et al. 2017), might pose a certain challenge for image registration (Mewes et al. 2007). This pattern of MD+ deviations was only seen in the preterm group suggesting higher variability in the volume, shape, and relative position of the ventricles in preterm babies.

The high spatial interindividual variability in the preterm group means that patterns of abnormality are not easy to detect on overlap maps; for example, a number of infants had significantly reduced MD (MD−) in the cerebellum which were not identified on the overlap maps. During the last trimester, the rapid cerebellar growth surpasses all other brain structures, and alterations to the maturational processes involved are a recognized vulnerability to neurocognitive difficulties in premature infants (Volpe 2009). The dramatic increase in cerebellar size is driven by the increase in cell density and their projections, with a considerable proportion of cerebellar neurogenesis and dendritic/axonal elaboration occurring postnatally (Ábrahám et al. 2001; Sathyanesan et al. 2019). The premature exposure to ex utero environment poses a significant risk for the typical progression of these maturational events and thus might represent a contributing factor to the MD− deviations observed in the preterm cerebellum.

Deviations in FA were less evident in both extent and spatial heterogeneity but even so the only regions where atypical values were detected in more than a few infants were in deep gray matter and corpus callosum. Imaging studies assessing the effect of prematurity on brain microstructure report only small to medium effects consistent with our findings of high interindividual variations between preterm infants. Despite that altered microstructure in the corpus callosum is one of the best documented findings in preterm infants (Counsell et al. 2008, 2013; van Kooij et al. 2012; Telford et al. 2017), here only approximately 10% of the preterm group had extreme deviations in this region. Combined with the highly variable interindividual MD+ deviations, this emphasizes that group-level studies might not capture fully the microstructural variation associated with prematurity.

Identifying modifiable risks early in development offers the potential to improve continued clinical management in preterm infants (Ment et al. 2009). Group analysis of MRI data have shown that microstructural disturbances in the preterm brain correlate with neurodevelopment at 2 years of age (Counsell et al. 2008; van Kooij et al. 2012; Ball et al. 2015; Duerden et al. 2015), and it is clear that MRI datasets encode valuable prognostic information. Nevertheless, the predictive power of MRI using group mean approaches is limited at an individual level. The current study suggests that this might be at least in part due to the extreme heterogeneity in the way prematurity shapes the brain. GPR might offer a tool for more precise interpretation of imaging changes and the possibility of personalized precision medicine for preterm infants.

## Limitations and Future Directions

Despite providing sensitivity to developmental and pathological changes in brain microstructure (Mori et al. 2002; Dean et al. 2013), the tensor model lacks specificity regarding the biological substrate driving variation (Jones et al. 2013). Thus, caution is essential when relating changes in tensor-derived metrics to maturational events. Several higher-order models have been developed to overcome some of these limitations and offer either measures with better biological interpretability (Zhang et al. 2012) or dealing with the complexity of the underlying microstructural composition (Tournier et al. 2004). Yet currently there is no model that is well suited for modelling both developing neonatal white and gray matter microstructure. Therefore, more work is essential to interrogate the biological bases of individual maturation differences in preterm neonates. Nonetheless, we emphasize the two main reasons why we choose DTI over more sophisticated diffusion models in this work. First and foremost, we sought to question the application of only first-order statistics (mean and median) in the study of biologically heterogeneous risk populations and to offer a novel approach, able to characterize the effects of prematurity on an individual infant level. Secondly, the minimal acquisition requirements necessary for fitting DTI will allow our results to be reproduced using datasets collected with less sophisticated acquisition parameters/hardware (e.g., clinical datasets collected in the past decade), and therefore we offer a method with high generalizability.

Importantly, model prediction was more accurate in the central gray matter and core WM tracts compared with the periphery of the brain, for example, the cortical ribbon. This is expected given the high intrasubject variability in the developing cortex. Novel surface-based registration algorithms driven by geometric features of cortical shape have been shown to offer better intersubject cortical alignment and lessen CSF contamination (Robinson et al. 2014; Bozek et al. 2018) and, thus, will with no doubt offer a powerful tool to address this challenge in future studies. Nonetheless, the application of GPR can be extended to investigate if the nature of an individual’s atypicality can inform personalized medicine, for example, the selection of educational or behavioral interventions which target the neural systems most affected.

## Conclusion

This study suggests that the highly variable spatial alterations seen in preterm infants needs to be accounted for in developing imaging strategies to understand mechanisms of injury and determine neurocognitive prognosis.

## Funding

The dHCP project was funded by the European Research Council under the European Union Seventh Framework Programme (FR/2007-2013)/ERC Grant Agreement no. 319456. The authors acknowledge infrastructure support from the National Institute for Health Research Mental Health Biomedical Research Centre at South London, Maudsley NHS Foundation Trust, King’s College London, the National Institute for Health Research Mental Health Biomedical Research Centre at Guys, and St Thomas’ Hospitals NHS Foundation Trust. The study was supported in part by the Wellcome Engineering and Physical Sciences Research Council Centre for Medical Engineering at King’s College London (grant WT 203148/Z/16/Z) and the Medical Research Council (UK) (grant MR/K006355/1). Support was also provided by EU-AIMS—a European Innovative Medicines Initiative. Flemish Research Foundation (grant number 12ZV420N to D.C.). Sir Henry Dale Fellowship jointly funded by the Wellcome Trust and the Royal Society (grant 206675/Z/17/Z to J.O.). G.M. received support from the Sackler Institute for Translational Neurodevelopment at King’s College London and from National Institute for Health Research (NIHR) Maudsley Biomedical Research Centre (BRC). The views expressed are those of the authors and not necessarily those of the NHS, the NIHR, the Department of Health. Medical Research Council Centre for Neurodevelopmental Disorders, King’s College London (grant MR/N026063/1 to J.O., A.D.E., and G.M.).

*Conflict of Interest:* The authors declare no conflict of interest.

## Supplementary Material

GPR-dwi-Suppl-CC-submission_bhaa069
