# Supplementary material for: Heterogeneity in Brain Microstructural Development Following Preterm Birth
Source: Cereb Cortex. 2020 Apr 18;30(9):4800–10. doi: 10.1093/cercor/bhaa069 (PMC7391275; doi:10.1093/cercor/bhaa069)
Supplement: GPR-dwi-Suppl-CC-submission_bhaa069 [file gpr-dwi-suppl-cc-submission_bhaa069.pdf]

Supplementary Materials

Dimitrova et al., 'Heterogeneity in brain microstructural development following preterm birth'

Supplementary Methods

Summary motion score

The amount of head motion detected for each infant was quantified using the total infant translation, rotation and the ratio of detected outliers following the procedure described in (Christiaens *et al.*, 2019). In brief, translation and rotation estimates were defined as the root-mean-squared forward difference of the respective translation/rotation parameter time series in their linearized Lie algebra representation. The ratio of outlier was defined as one minus the mean slice weight across the dataset. To compute one summary index of total head motion detected during the dMRI sequence for every infant, each of these three metrics was first normalised to its respective median across the entire dHCP dataset, consisting of 695 scanning session (at the time of publication). Finally, the summary motion measure was calculated as the root-mean-squared value across the rescaled translation, rotation and outlier ratio.

Vargha and Delaney's A effect size estimate

Vargha and Delaney's A ranges from 0 to 1, with A = 0.5 indicating two groups are stochastically equal, A = 1 indicating that the reference group is dominant over the second, and A = 0 reporting that the second group is dominant over the reference group. Therefore, small effect size is considered when $0.56 > A > 0.64$ or $0.44 > A > 0.34$, medium effect size, when $0.64 > A > 0.71$ or $0.34 > A > 0.29$ and large effect size when $A \geq 0.71$ and $A \leq 0.29$.

Supplementary Figures

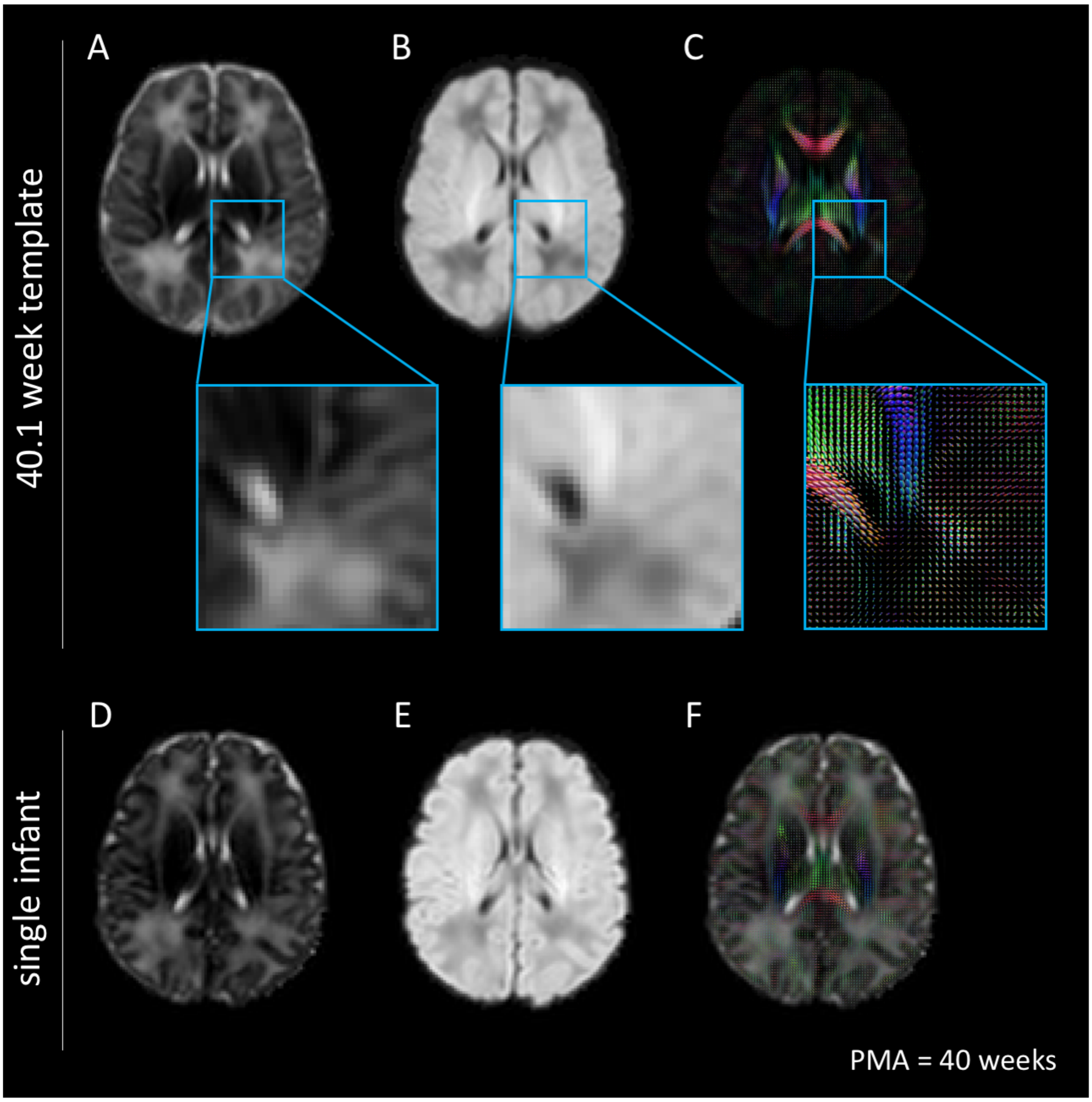

Supplementary Figure 1. Fluid (A and D) and tissue (B and E) component volume fraction maps (grayscale, scaled identically) and tissue ODF surface renderings (C and F) for the 40.1-week neonatal template (A-C) and for an individual infant scanned at 40 weeks PMA (D-F). A-C also highlight the parietal periventricular WM crossroad, a region characterized by high fluid and low tissue density during early development.

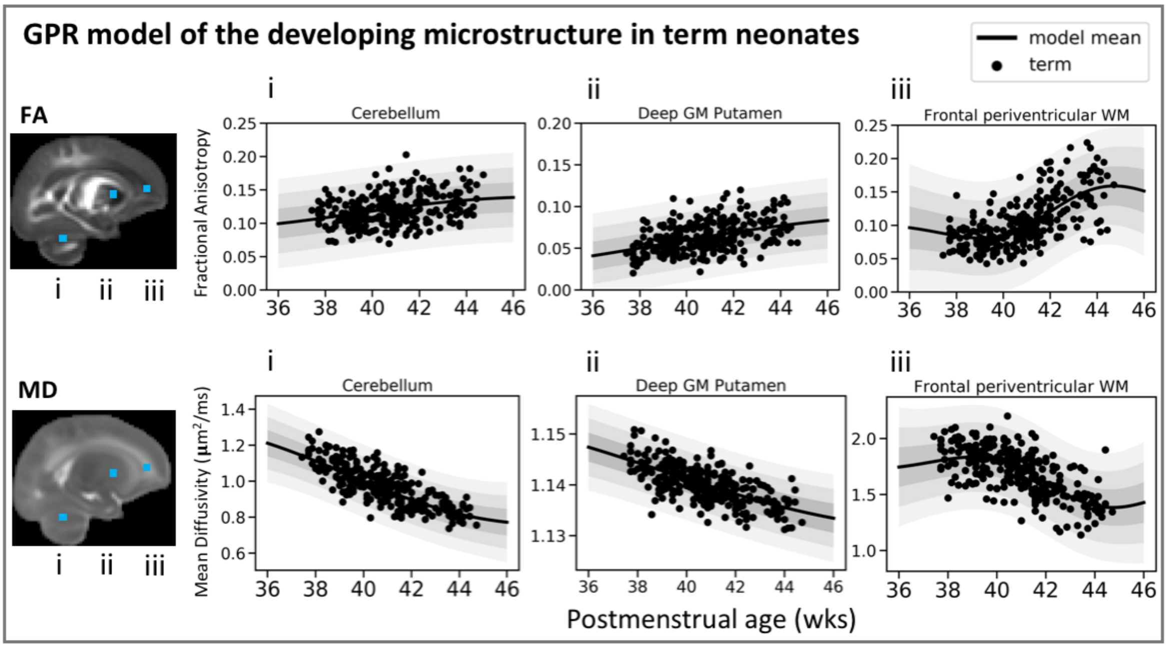

Supplementary Figure 2. Modelling developing microstructure in the term neonatal brain using gaussian process regression (GPR). The predicted developmental trajectories are plotted for three randomly selected brain voxels located in the cerebellum (i), deep GM putamen (ii) and frontal periventricular WM (iii). The relative location of the voxels is highlighted with blue squares on the observed mean FA and MD maps. Plots show the model mean (thick black) ±1 (dark grey), ±2 (light grey) and ±3 (lighter grey) standard deviations from the predicted mean and the diffusion values extracted for these voxels for all 270 term infants.

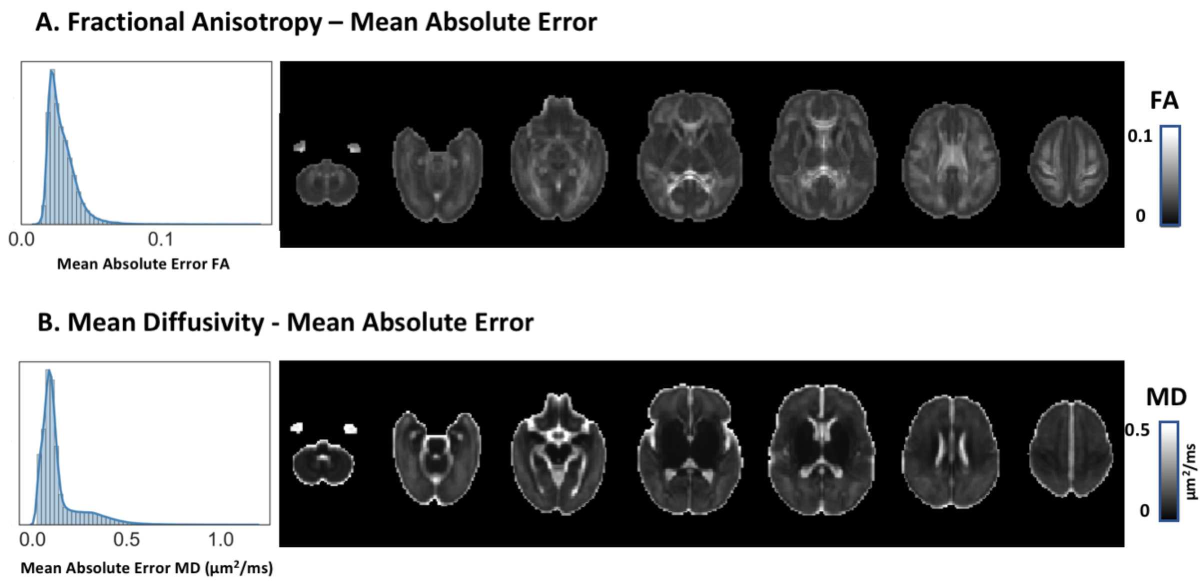

Supplementary Figure 3. Mean absolute error (MAE) for Fractional Anisotropy (A) and Mean Diffusivity (B) model prediction. Histograms show the distribution of MAE across the MAE map and the 3D maps are presented on the right.

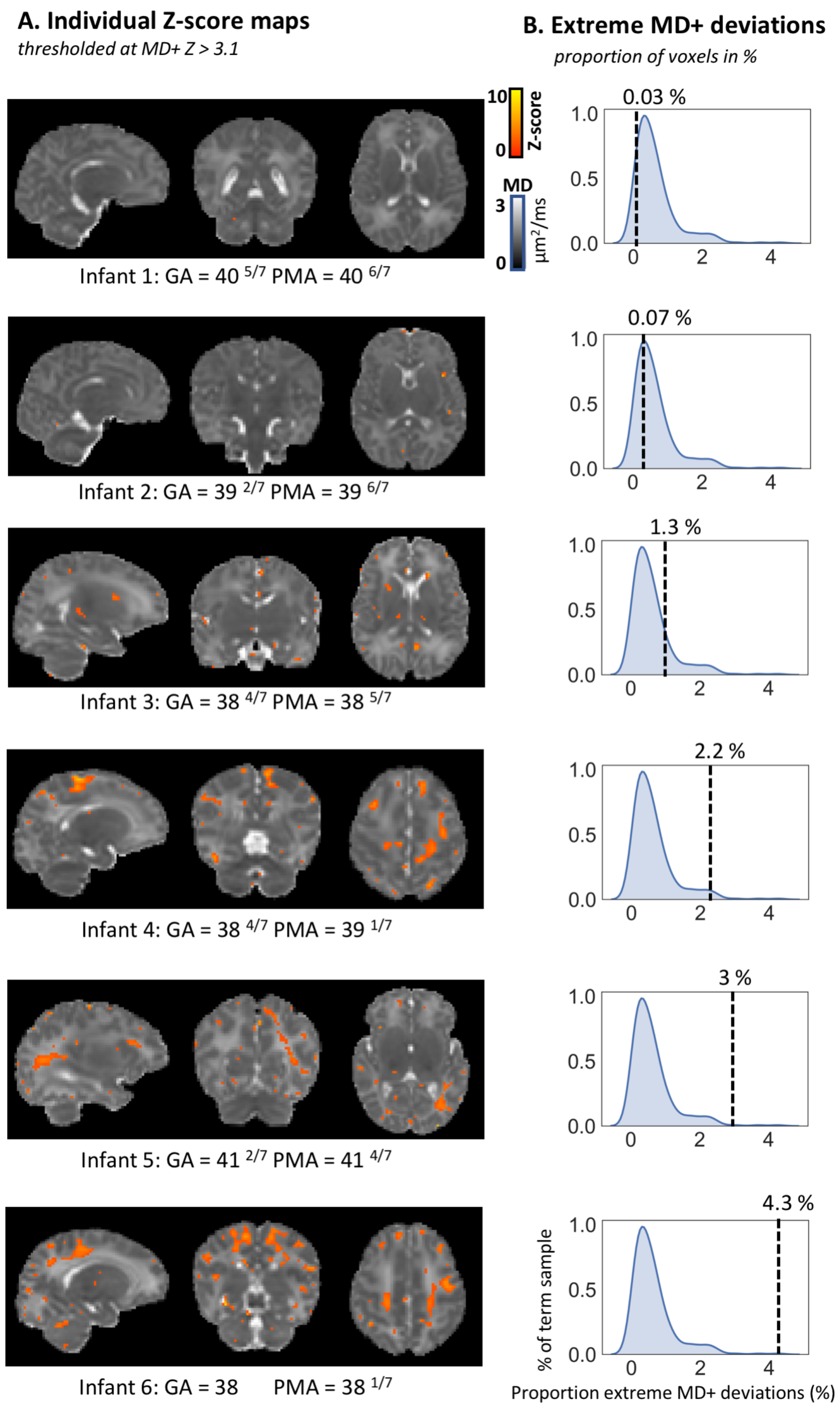

Supplementary Figure 4. Heterogeneity in typical microstructural development in the term born neonatal brain. Individual MD+ deviations (Z-score maps thresholded at Z>3.1) are shown for 6 term-born infants depicting the unique spatial patterns observed within the term born sample (**A**). The density plots (**B**) indicate where is the overall brain proportion of

extreme deviations for this infant in relation to the rest of the term sample. Infants were selected to show different overall proportion of extreme deviations. Infant 1 had a relatively low atypicality index (0.03% of voxels), while infant 6 had a relatively high index (4.3%) of voxels deviating from the model. The GA at birth and PMA at scan (in weeks $^{+days}$) are shown for each infant.

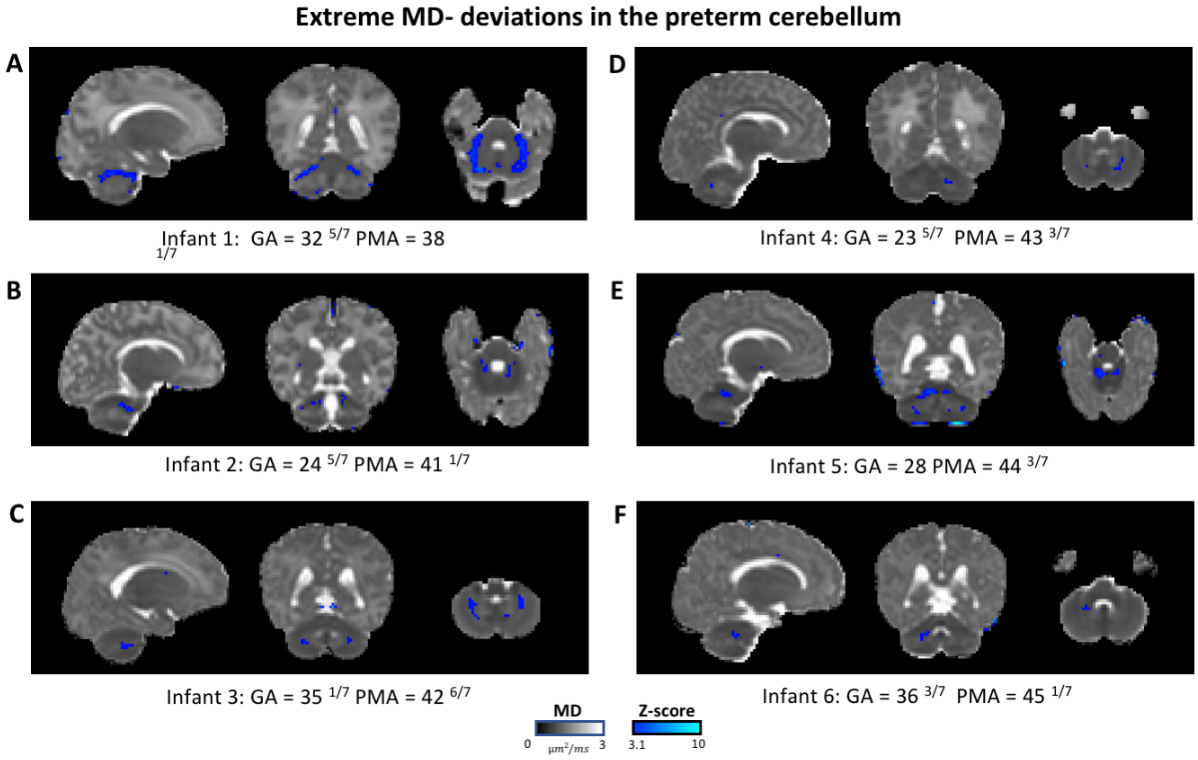

Supplementary Figure 5. **Extreme negative MD deviations from the normative model in the preterm cerebellum.** Extreme deviation maps (Z > 3.1) for 6 preterm infants (**A – F**) are overlaid onto subjects' individual MD maps. GA at birth and PMA at scan are also given for every infant (in weeks $^{+days}$).

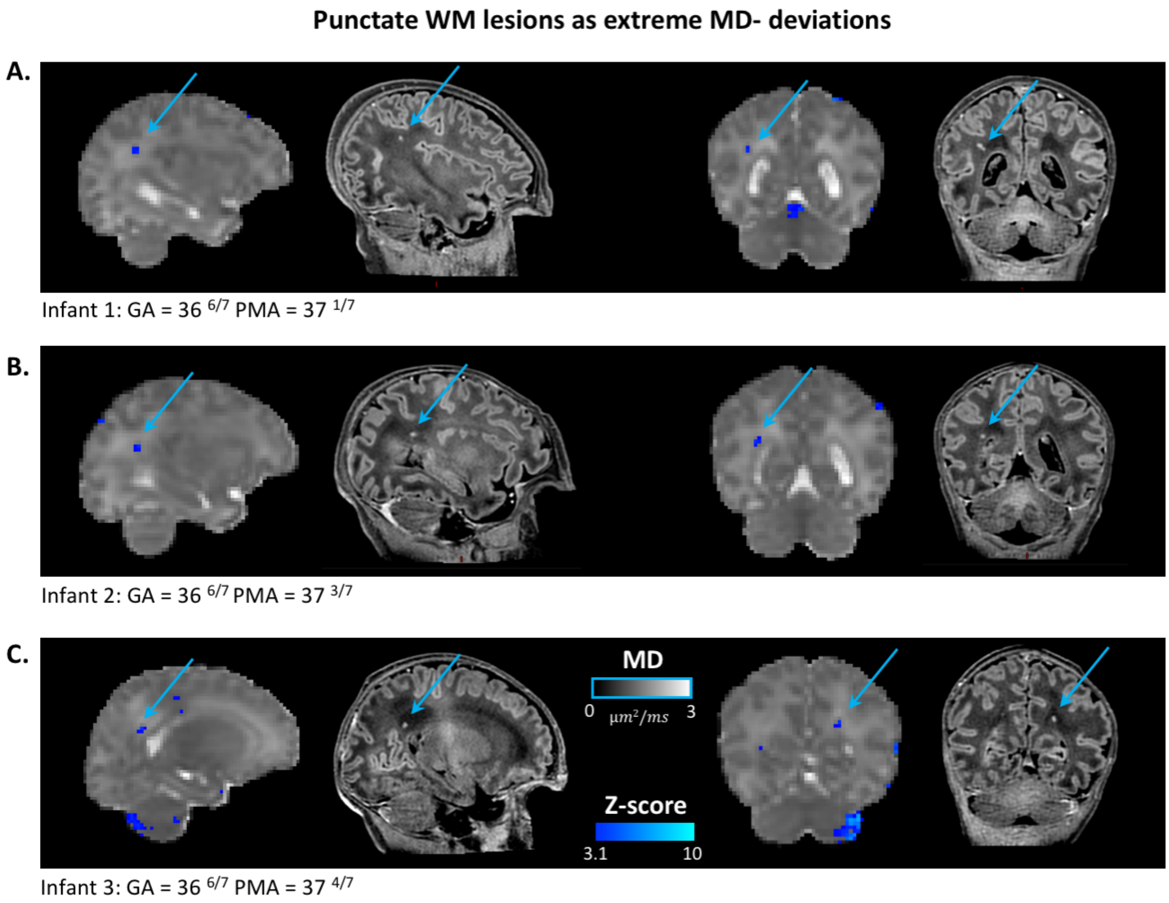

Supplementary Figure 6. Punctate White Matter lesions (PWMLs) detected by the GPR model as extreme negative MD deviations. The individual extreme MD– maps for 3 preterm infants (**A - C**) are shown overlaid onto the subject's MD map on both sagittal and coronal view. T_1-weighted images are also shown for these subjects (note the original contrast has been adjusted so that PWMLs are easier to detect). PWMLs are indicated with blue arrows on the MD maps and the T_1-weighted images. GA at birth and PMA at scan are also given for every infant (in weeks $^{+days}$).

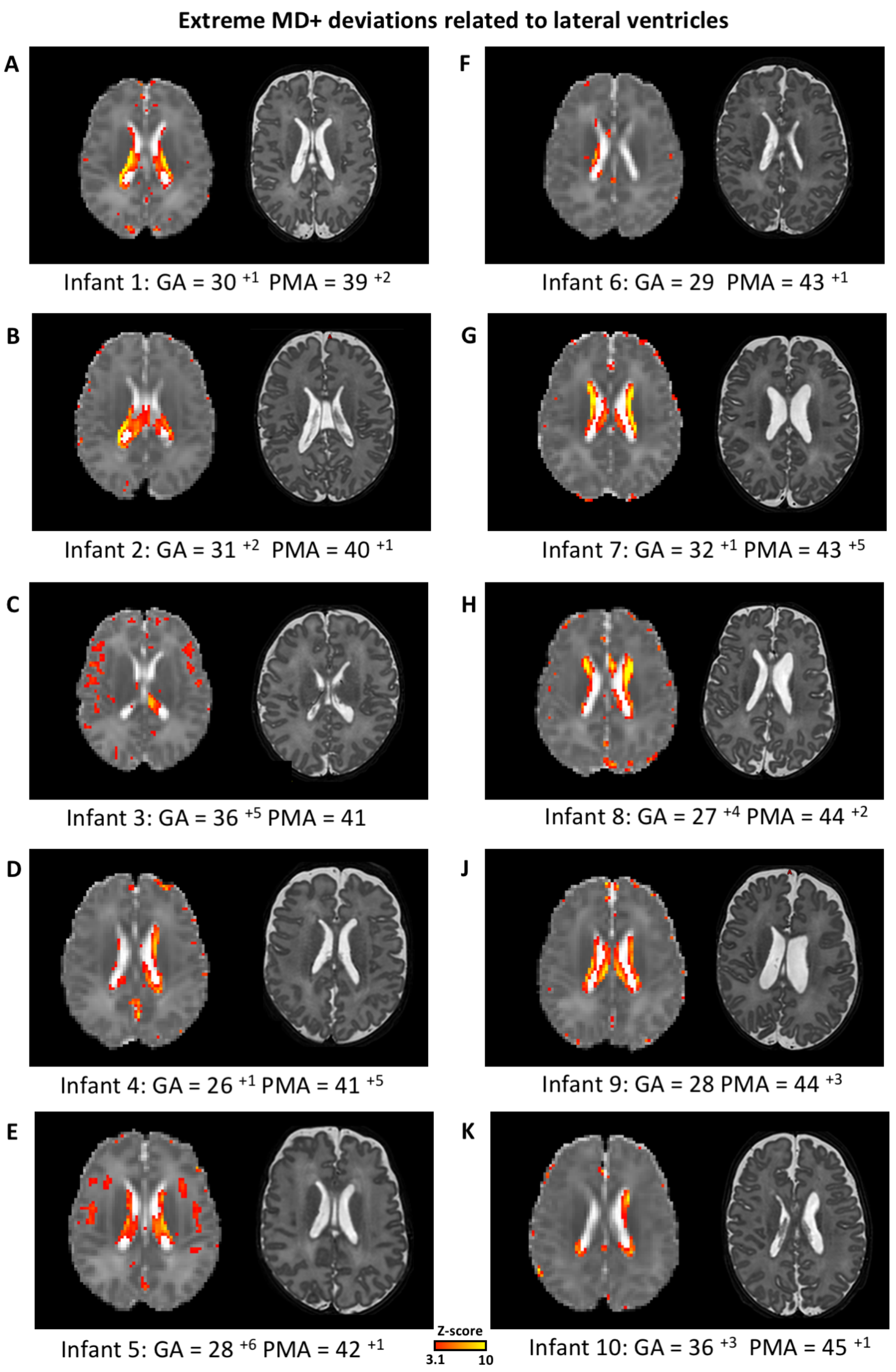

Supplementary Figure 7. Extreme positive MD deviations ($Z > 3.1$) in the preterm sample in voxels around the ventricles. Individual extreme Z-score maps for 10 preterm infants (**A-K**) are overlaid onto the individual MD map complimented by the corresponding T$_2$-weighted image. GA at birth and PMA at scan are also given for every infant (in weeks $^{+days}$).

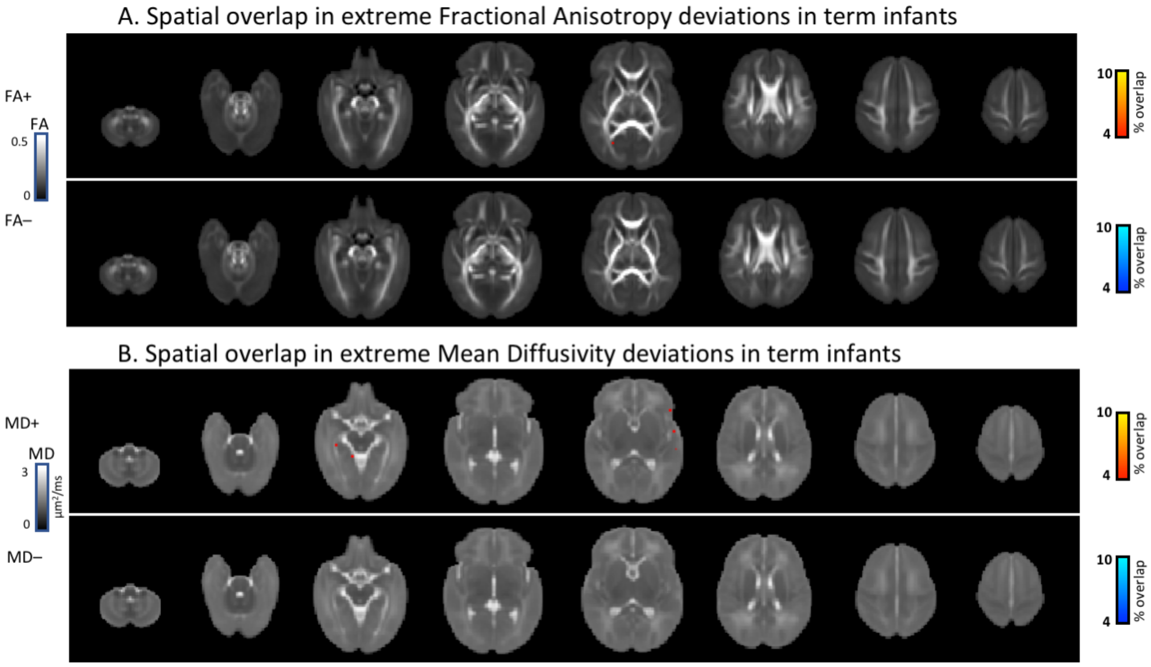

Supplementary Figure 8. Percentage spatial overlap of extreme positive and negative FA and MD deviations across the term-born neonatal brain. Spatial overlap Fractional Anisotropy (A) and Mean Diffusivity (MD) maps, thresholded at 4% (11 out of 270 term infants showed extreme deviations) overlaid onto mean FA and MD maps, averaged across the term-born sample.

Supplementary Tables

Supplementary Table 1. Spearman correlation between GA at birth and BSID-III scores at 18 months. In bold, significant associations after multiple comparison correction.

	**Gestational Age (GA) at birth**		
	**Whole sample** *n = 257*	**Term group** *n = 210*	**Preterm group** *n = 47*
**Motor**	0.12 *p = .12*	0.10 *p = .39*	0.30 *p = .09*
**Language**	0.03 *p = 1*	0.10 *p = .39*	0.11 *p = .46*
**Cognition**	0.03 *p = 1*	0.09 *p = .39*	***0.38 p < .05***

Note: p-values presented at corrected for multiple comparison using Bonferroni-Holm method

Supplementary table 2. Spearman correlations between index of multiple deprivation (IMD) and BSID-III scores at 18 months. In bold, significant associations after multiple comparison correction.

	**Index of Multiple Deprivation (IMD)**		
	**Whole sample** *n = 254*	**Term group** *n = 210*	**Preterm group** *n = 44*
**Motor**	-0.02 *p = .76*	-0.02 *p = .81*	-0.09 *p = .95*
**Language**	-0.11 *p = .18*	-0.06 *p = .77*	-0.17 *p = .77*
**Cognition**	***-0.25 p < .005***	***-0.26 p < .005***	-0.11 *p = .95*

Note: p-values presented at corrected for multiple comparison using Bonferroni-Holm method

Supplementary Table 3. Spearman correlations between extreme deviations from the model and BSID-III scores at 18 months. In bold, significant associations after multiple comparison correction.

	**Whole sample** *n = 257*			**Term group** *n = 210*			**Preterm group** *n = 47*		
	**Motor**	**Lang.**	**Cog.**	**Motor**	**Lang.**	**Cog.**	**Motor**	**Lang.**	**Cog.**
**FA+**	0.01 *p = 1*	-0.05 *p = 1*	-0.04 *p = 1*	0.00 *p = 1*	-0.03 *p = 1*	-0.01 *p = 1*	-0.35 *p = .13*	-0.16 *p = .59*	-0.19 *p = .59*
**FA–**	-0.09 *p = 1*	-0.04 *p = 1*	-0.04 *p = 1*	-0.02 *p = 1*	0.00 *p = 1*	0.01 *p = 1*	0.04 *p = 1*	**-0.46** ***p < .05***	**-0.47** ***p < .05***
**MD+**	-0.14 *p = 0.3*	-0.01 *p = 1*	-0.11 *p = .6*	-0.10 *p = 1*	-0.02 *p = 1*	-0.13 *p = .65*	-0.35 *p = .13*	-0.32 *p = .16*	**-0.40** ***p < .05***
**MD–**	-0.09 *p = 1*	-0.16 *p = .1*	-0.14 *p = .23*	-0.03 *p = 1*	-0.11 *p = 1*	-0.12 *p = .8*	-0.28 *p = .22*	**-0.40** ***p < .05***	-0.29 *p = .23*

Note: p-values presented are corrected for multiple comparison using Bonferroni-Holm method
